# Mapping the baseline prevalence of lymphatic filariasis across Nigeria

**DOI:** 10.1186/s13071-019-3682-6

**Published:** 2019-09-16

**Authors:** Obiora A. Eneanya, Claudio Fronterre, Ifeoma Anagbogu, Chukwu Okoronkwo, Tini Garske, Jorge Cano, Christl A. Donnelly

**Affiliations:** 1 MRC Centre for Global Infectious Disease Analysis, Department of Infectious Disease Epidemiology, Imperial College London, London, UK; 2 Faculty of Infectious and Tropical Diseases, London School of Hygiene and Tropical Medicine, London, UK; 3 Federal Ministry of Health, Abuja, Nigeria; 4 Department of Statistics, University of Oxford, Oxford, UK

**Keywords:** Lymphatic filariasis, Machine learning, Antigenaemia, Microfilaraemia

## Abstract

**Introduction:**

The baseline endemicity profile of lymphatic filariasis (LF) is a key benchmark for planning control programmes, monitoring their impact on transmission and assessing the feasibility of achieving elimination. Presented in this work is the modelled serological and parasitological prevalence of LF prior to the scale-up of mass drug administration (MDA) in Nigeria using a machine learning based approach.

**Methods:**

LF prevalence data generated by the Nigeria Lymphatic Filariasis Control Programme during country-wide mapping surveys conducted between 2000 and 2013 were used to build the models. The dataset comprised of 1103 community-level surveys based on the detection of filarial antigenemia using rapid immunochromatographic card tests (ICT) and 184 prevalence surveys testing for the presence of microfilaria (Mf) in blood. Using a suite of climate and environmental continuous gridded variables and compiled site-level prevalence data, a quantile regression forest (QRF) model was fitted for both antigenemia and microfilaraemia LF prevalence. Model predictions were projected across a continuous 5 × 5 km gridded map of Nigeria. The number of individuals potentially infected by LF prior to MDA interventions was subsequently estimated.

**Results:**

Maps presented predict a heterogeneous distribution of LF antigenemia and microfilaraemia in Nigeria. The North-Central, North-West, and South-East regions displayed the highest predicted LF seroprevalence, whereas predicted Mf prevalence was highest in the southern regions. Overall, 8.7 million and 3.3 million infections were predicted for ICT and Mf, respectively.

**Conclusions:**

QRF is a machine learning-based algorithm capable of handling high-dimensional data and fitting complex relationships between response and predictor variables. Our models provide a benchmark through which the progress of ongoing LF control efforts can be monitored.

## Introduction

LF is thought to be endemic in large parts of Nigeria [1]. Although endemicity mapping is mostly completed at district level nationwide [2], some districts in the North-East remain unmapped mainly due to security issues. For mapped areas, there is a need to further understand intra-district heterogeneity in prevalence. Modelling has shown that the success of control programmes to interrupt LF transmission highly relied upon the intensity of transmission prior to the scale-up of MDA interventions [3]. Therefore, knowing the spatial heterogeneity in the intensity of infection throughout implementation areas, as opposed to simply endemic/non-endemic classification, would enable control programmes to identify areas which may require enhanced interventions as they approach the endgame in the elimination pathway. Furthermore, producing maps based on a modelling approach serves as a tool to validate endemicity maps that are already in use for control programmes and potentially identify any discrepancies in endemicity classifications.

Control of LF is largely based on MDA interventions, whereby entire endemic populations are treated with repeated rounds of antifilarial medications. Prior to the implementation of this interventions it was necessary to determine the endemicity status of the geographical areas to be treated [4]. Mapping surveys relied upon the detection of circulating filarial antigens in blood samples of adults from selected communities using a rapid ICT card [5]. However, prior to the existence of ICT tests, LF diagnosis was based on the detection of circulating filarial worm, Mf, by microscopic examination of thick blood smears [6]. These tools are key to LF control programmes because they inform decisions regarding endemicity status, allow for monitoring control interventions and ultimately provide the necessary evidence of interruption of infection transmission.

Although Rapid Assessment of the Geographical Distribution of Bancroftian Filariasis (RAGFIL) surveys have been used to generate initial estimates of the burden of LF [7, 8], these estimates have been improved upon by the use of geostatistics. Geostatistical modelling has enabled the prediction of infection prevalence in unsampled locations across large geographical areas using a suite of potential disease drivers such as climate, environmental and demographical data [9–15]. Models that take into account the spatial structure of the infection distribution are commonly used both in frequentist [16] and Bayesian [17] modelling frameworks for prevalence mapping. The importance of accounting for spatial effects in prevalence models has been previously explored [18–20], and methods for handling spatially correlated data have been suggested [16, 17]. Generalised linear models with spatially correlated random effects, otherwise known as generalised linear spatial models (GLSMs), are widely used to fit binomial data with spatial structure [18]. Despite this being the method of choice for modelling prevalence data for a variety of diseases including schistosomiasis [21], LF [10] and malaria [22], a major drawback of GLSMs is their limitations to handle high-dimensional, non-linear and collinear predictors and response datasets [23]. Machine learning based algorithms have proven to be powerful tools to handle complex relationships between continuous and binary data and independent covariates [24, 25], but little is known about their performance when it comes to modelling binomial data obtained through randomised surveys [26].

In this study, a model was trained based on baseline prevalence data collected through mapping surveys conducted across Nigeria and a suite of environmental and demographic data using a machine learning algorithm, Quantile Regression Forest (QRF). Then, the trained model was used to predict the prevalence and related uncertainty for unsampled locations based on the selected predictors.

## Methods

### Lymphatic filariasis data

Community-level prevalence data (both ICT and Mf) collected during nationwide mapping surveys conducted by the Nigeria Lymphatic Filariasis Control Programme from 2000–2013 was used in this analysis. Also, historical data, mostly surveys based on parasitological diagnosis (Mf detection), publicly available and assembled by the Global Atlas of Helminth Infections [27] were included. All surveys were conducted prior to the implementation of MDA interventions. Up to two communities were surveyed by local government area (LGA) during nationwide mapping. The LGA corresponds to the second administrative level for Nigeria and is considered the geographical area for the implementation of control interventions, thus called the implementation unit (IU).

Within each IU at least one sample village was randomly selected for survey and a buffer zone of at least 50 km separated pairs of sample villages. LF endemicity was estimated by testing for filarial antigenemia in peripheral blood using rapid ICT. For sample villages recording ≥ 1% prevalence, the entire IU is considered endemic for LF. IUs which have frequent reports of hydrocele and lymphedema are strongly suspected to have high endemicity for LF. Such villages are thus identified as sentinel sites for evaluating control programmes and, in addition to ICT card test, LF burden is estimated by Mf load in venous blood collected during night-time surveys. The timing of blood collection coincides with the appearance of Mf in blood, known as nocturnal periodicity. The sampling methodology for the LF surveys has been described in greater detail elsewhere [28]. The mapping methodology described above, however, does not apply to urban LF infections mainly due to the differences in LF vectors and living conditions between urban and rural areas [29].

**Fig. 1:**
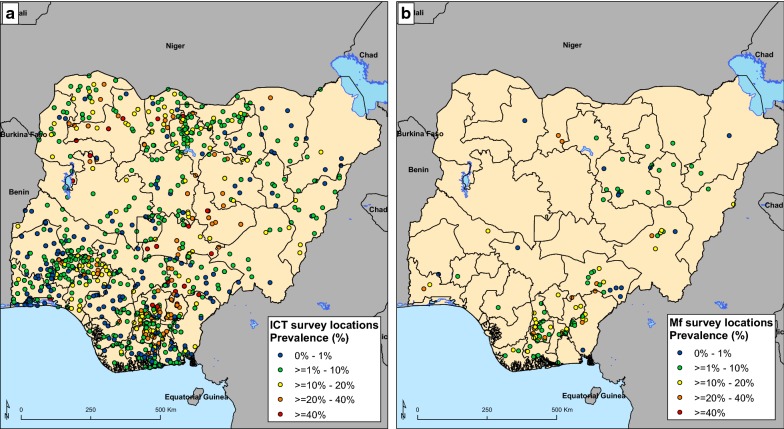
Location of study sites in Nigeria showing the prevalence of lymphatic filariasis. **a** Immunochromatographic test (ICT). **b** Microfilaria (Mf)

For this analysis, we had 1103 ICT and 184 Mf prevalence estimates (Fig. 1). These were all pre-intervention data testing 142,881 survey participants, 15 years or older. In building the model, we utilized both ICT and Mf observed prevalence, creating a new covariate ‘diagnostic type’ (an indicator variable) to assess the effects of diagnostic method on the spatial distribution of LF. Mean observed prevalence for ICT and Mf were 9.5% and 5.0%, respectively.

The spatial correlation in the observed LF prevalence dataset was measured using a variogram analysis. The variogram gives a measure of the variability between pairs of datapoints [18]. This is important as it was used to assess the degree of spatial autocorrelation that remains on the residuals of the fitted models. Figure 1 shows the distribution of survey locations for ICT and Mf respectively.

### Climate and environmental predictors

Continuous gridded maps of climate, topography, vegetation and land use for Nigeria were obtained from different sources. All variables considered had biologically plausible association to LF prevalence. In total, 17 environmental variables were considered in the construction of this model. Precipitation and temperature variables processed from the WorldClim database were all long-term (1950–2000) averages of data from weather stations distributed across the world [30]. A detailed description of these predictors and their processing is provided in our previous work [28]. All input grids raster covariates were resampled to a common spatial resolution of 1 × 1 km^2^ using the nearest-neighbour algorithm [31].

### Quantile regression forest models

QRF is an extension of Random Forest (RF) and is adept at handling large, complex datasets [32]. RF algorithm is an ensemble learning method for classification and regression based on the construction of regression decision trees. This machine learning-based algorithm has proven to outperform other approaches under similar modelling problems [33, 34]. Briefly, trees are grown through recursive partitioning into binary splits from a primary root node which contains all data. For each split, a random subset of predictor variables (approx. one third) is used to grow new root nodes. Each partition contains a different random bootstrapped sample (approx. two thirds) of the dataset. Using bootstrapped samples avoids the problem of overfitting in RF models [35]. This process is repeated until a terminal node is reached, and the average of all the trees is used to make predictions. The response variables not selected during binary node splits, known as the ‘out-of-bag’ cases, are used to evaluate the predictive accuracy of the model and generating estimating the variable importance.

In RF models (for regression), only the mean of the bootstrapped response variable is considered when splitting/growing trees and for quantile determination [34]. All other features of the response variable of possible interest are neglected. QRF was thus developed to consider all the values in the response variable for splitting and quantile determination [32, 36, 37]. Therefore, QRF enables the estimation of any quantile from the entire posterior conditional distribution for a modelled outcome. Accounting for all the features of the response variable is thought to give a more complete picture of the dataset and resulting predictions [38]. The RF and QRF algorithm uses bagging to randomly resample the training dataset (with replacement of original data) and builds a forest of trees, whereas boosted regression trees use boosting to randomly resample training dataset (without replacement) and builds a sequence of trees with each added tree focussing on poorly fitted nodes.

In this work, an RF model is initially fitted to tune parameters for use in the QRF. Here a 10-fold internal cross-validation was performed and repeated 5 times on empirical logit-transformed infection prevalence and set of predictors. The empirical logit-transformation of infection prevalence was weighted by number of individuals examined using the following formula:

$${\tilde{Y}_{i} = \log \left( {\frac{{Y_{i} + \frac{1}{2}}}{{m_{i} - Y_{i} + \frac{1}{2}}}} \right)}{:} \quad i = 1, \ldots , n$$

where Y_i_ is the observed number of people infected at location *i* and $m_{i}$ is the number of people examined.

### This tuning process informs an optimum number of predictor variables to be considered at each node split

Using the optimal number of predictors yielded above, a QRF model was then constructed. For the QRF model, data were partitioned into two, with a random subset of 25% of the complete dataset retained for model validation and the remaining 75% used to train the model. The mean, median and prediction intervals estimates were obtained and projected over a continuous geographical space at a spatial resolution of 5 × 5 km. The RF and QRF models were implemented using the *randomForest* [39] and *quantregForest* [32] packages, respectively, in R (v.3.3.2) [40]. Predictive maps were exported into ArcGIS v10.3 for preparing map layouts [41].

Model evaluation was performed using the validation dataset based on the root mean squared error (RMSE) and R-squared scores (*R*^2^). Variable importance was represented by percentage increase in mean square error (%IncMSE). The %IncMSE is estimated with out-of-bag cross-validation as a result of a variable being permuted (values randomly shuffled). The difference between the calculated mean square errors is then averaged over all trees and then normalised by the standard deviation of the differences [42]. If a predictor is important in the model, then assigning other values for that predictor randomly should have a negative influence on prediction, thereby resulting in a higher %IncMSE value. To check for the existence of spatial structure on the data not captured by the predictors after implementing the QRF model, an empirical variogram was calculated based on the residuals of the QRF model. The Pearson’s correlation coefficient was calculated between pairs of observed and predicted ICT and Mf values. Results were presented as 95% prediction intervals and *P*-values.

From the final predicted prevalence maps, the number of people infected with LF was calculated. By overlaying the prevalence predictions on 2010 gridded population density estimates obtained from the WorldPop Africa repository [43], on a cell-by-cell basis, estimates for the infected population in each cell was generated. Population density data available for Nigeria were for the years 2006, 2010, 2015 and 2020. As the survey data used for building the models spanned from 2000–2013, we estimated the population infected based on population density estimates of 2010. All cells were summed up to get estimates for mainland Nigeria. This analysis was calculated using the Zonal Statistics function within the Spatial Analyst Tool in ArcGIS v10.3 [41].

## Results

Analysis for the models was performed using a total of 1287 site-level infection prevalence surveys for ICT (1103 surveys) and Mf (184 surveys) tests respectively as shown in Fig. 1.

### Variogram analysis

**Fig. 2:**
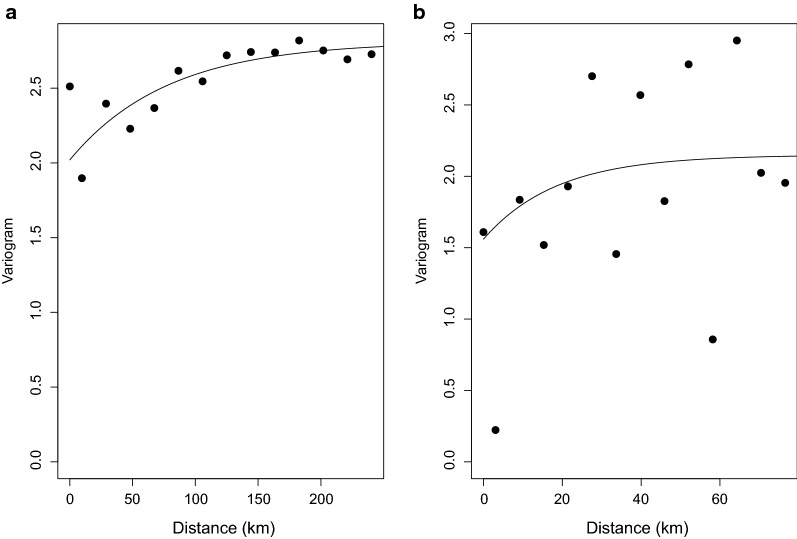
Variogram plot showing the spatial correlation in observed LF data. **a** Immunochromatographic test (ICT). **b** Microfilaria (Mf). The empirical variogram is represented by black dots; the theoretical variogram is represented by a solid line

The results of the variogram analysis (Fig. 2) indicate that there is significant spatial correlation in the observed ICT prevalence data. The range of spatial correlation is about 250 km after which the points start flattening out, an indication of the limit of spatial correlation between datapoints. Conversely, for Mf prevalence, there is limited evidence of spatial correlation, even at shorter distances.

### Variable importance plot

**Fig. 3:**
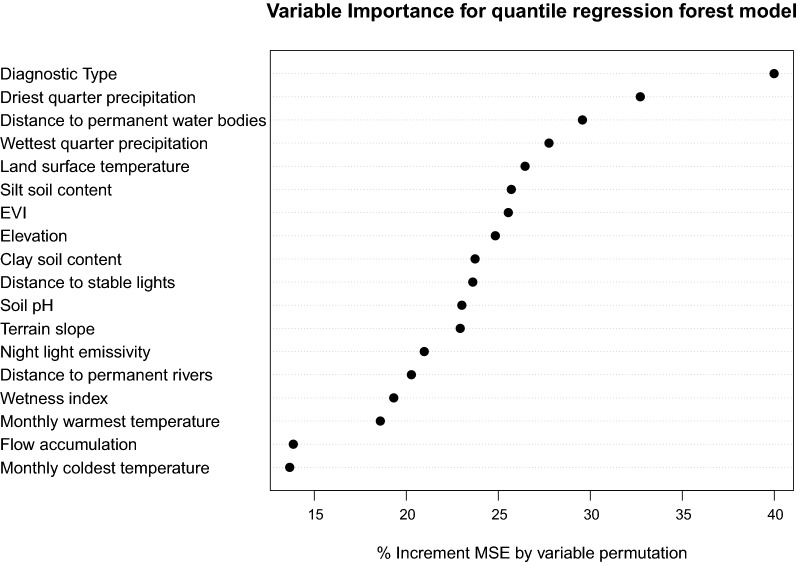
Variable importance for the trained model analysing both immunochromatographic test and microfilaria data simultaneously

Figure 3 shows the variable importance plot of the QRF model trained using LF prevalence data. Here, %IncMSE shows that diagnostic type, precipitation in the driest and wettest quarter, distance to permanent water bodies and land surface temperature were the 5 most important predictors for constructing our model.

### Predicted ICT and Mf prevalence

Predicted prevalence estimates were projected on the map of mainland Nigeria based on a suite of climate and environmental predictors at a spatial resolution of 5 × 5 km. RMSE and R-square values for the model were 1.24 and 0.40, respectively.

**Fig. 4:**
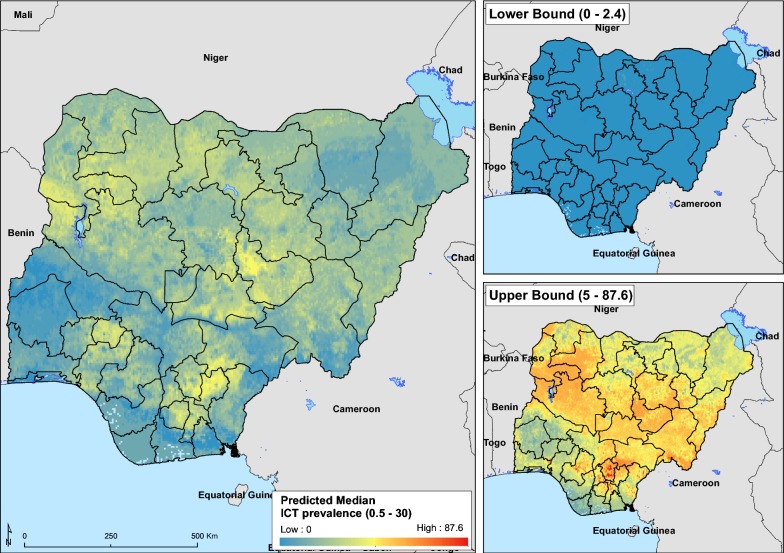
Median and 95% prediction interval of the immunochromatographic test (ICT) prevalence prediction

The maps presented in Fig. 4 is the predicted median and upper and lower bounds of ICT prevalence of LF in Nigeria. The median ICT map suggests a high prevalence of LF primarily in three out of six regions in Nigeria, namely North-West, North-Central and South-East of Nigeria. Also, transmission appears to be high in the South-West state of Ekiti.

**Fig. 5:**
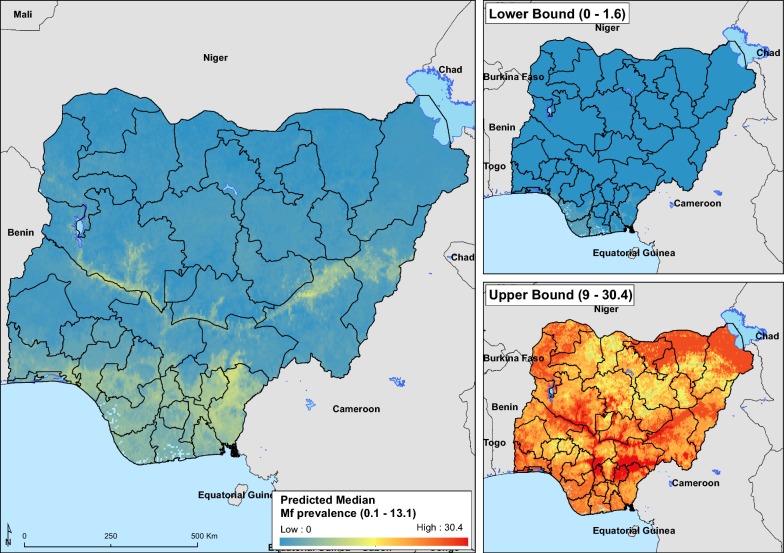
Median and 95% prediction interval of the microfilaria (Mf) prevalence prediction

The median Mf maps in Fig. 5 shows a distinct spatial pattern of LF, predicting a higher Mf prevalence in much of the southern region of Nigeria and along the course of Niger and Benue rivers. All regions (except the North-West) had predicted prevalence exceeding 1% for Mf.

### Validation of predictive models

**Fig. 6:**
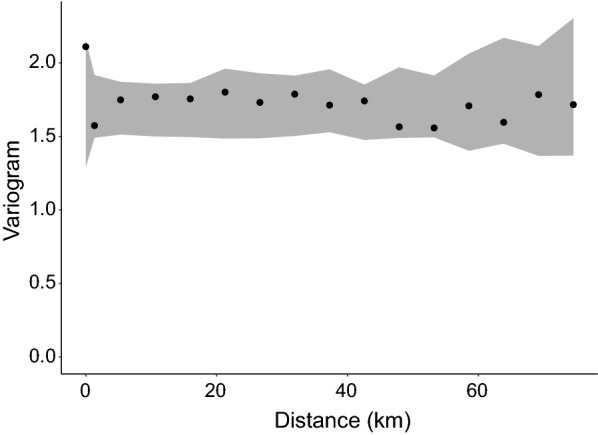
Empirical variogram of the residuals resulting from trained Quantile Regression Forest model

The variogram fitted on the residuals demonstrates that the trained QRF model, despite this not being a spatially explicit model, was able to capture, through some of the spatially varying predictors, the spatial structure in the observed ICT and Mf prevalence (Fig. 6). When exploring the correlation between observed and predicted ICT and Mf prevalence, there was a significant positive correlation: Pearson’s coefficient of 0.63 (95% CI: 0.57–0.67) and 0.51 (95% CI: 0.37–0.60) between observed and fitted values for ICT and Mf prevalence respectively.

**Fig. 7:**
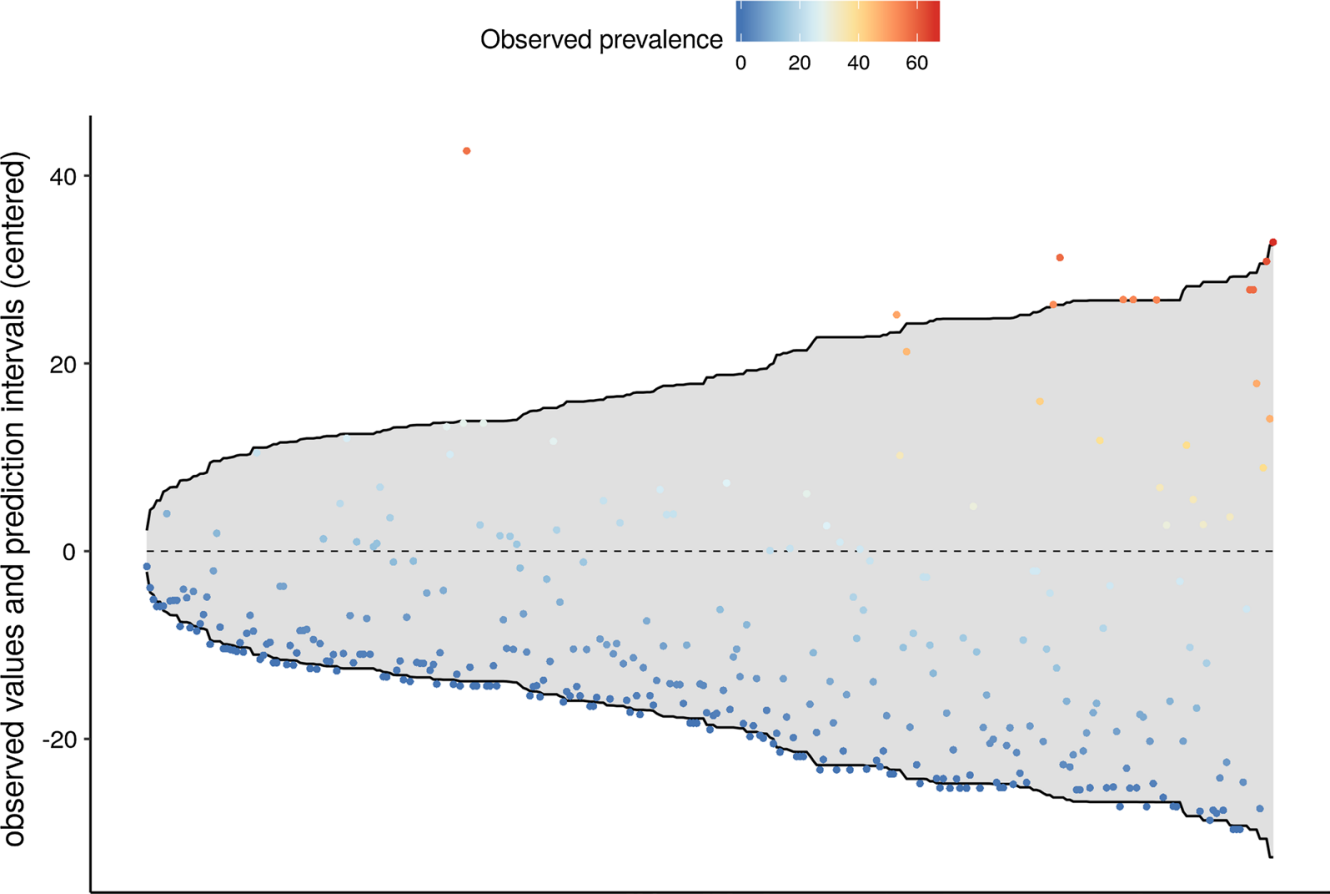
Cross-validation of the predicted lymphatic filariasis prevalence using a subsample of 25% of the observed data

Also, cross-validation of the predicted LF prevalence using a subsample of 25% of the observed data was performed (Fig. 7). Predictive intervals (shadow area) have been centred and observed prevalence for held-out subsample plot on it. 75.2% of the surveys fall within the prediction intervals. In Additional file 1: Figure S1, predicted prevalence values are plotted against observed prevalence. Those plotted in red had observations outside of the 95% prediction intervals while those plotted in blue had observations inside the 95% prediction intervals. Many of the observations plotted in red had 0% observed prevalence.

### Estimating population infected with lymphatic filariasis

The mean human population infected with LF is estimated to be 8.7 million and 3.3 million for ICT and Mf respectively (Table 1). This amounts to a national prevalence of 5.3% for ICT and 2.0% for Mf. Total national population for Nigeria for 2010 was derived from gridded population density estimates from the WorldPop repository [43, 44].

**Table 1:** Estimated number of people infected with lymphatic filariasis prior to MDA using 2010 population estimates

Region	State	Estimated no. of people infected with LF (% prevalence of Mf)	Estimated no. of people infected with LF (% prevalence of ICT)	Total population
North-Central				
	Benue	69,433 (1.4)	266,245 (5.5)	4,853,000
	Kogi	77,475 (2.0)	182,144 (4.7)	3,838,000
	Kwara	45,953 (1.6)	115,596 (4.1)	2,852,000
	Nasarawa	42,696 (2.0)	227,455 (10.6)	2,151,000
	Niger	85,667 (1.9)	282,768 (6.2)	4,538,000
	Plateau	71,432 (2.0)	364,561 (10.0)	3,659,000
	FCT	24,525 (1.6)	125,614 (8.2)	1,537,000
Subtotal		417,191 (1.8)	1,912,940 (8.2)	23,428,000
North-East				
	Adamawa	76,843 (2.3)	209,067 (6.4)	3,272,000
	Bauchi	25,366 (0.5)	332,294 (6.3)	5,257,000
	Borno	59,503 (1.3)	205,103 (4.3)	4,752,000
	Gombe	43,744 (1.6)	149,619 (5.4)	2,773,000
	Taraba	70,051 (2.6)	159,935 (6.0)	2,657,000
	Yobe	27,115 (1.0)	209,281 (7.9)	2,652,000
Subtotal		302,622 (1.4)	1,265,299 (5.9)	21,363,000
North-West				
	Jigawa	48,972 (1.0)	343,852 (6.8)	5,054,000
	Kaduna	17,790 (0.3)	385,812 (5.6)	6,927,000
	Kano	91,093 (0.8)	717,153 (6.7)	10,765,000
	Katsina	58,317 (0.9)	477,381 (7.3)	6,550,000
	Kebbi	54,026 (1.4)	395,222 (10.5)	3,758,000
	Sokoto	42,794 (1.0)	275,762 (6.7)	4,137,000
	Zamfara	44,103 (1.2)	597,723 (16.2)	3,689,000
Subtotal		357,095 (0.9)	3,192,945 (7.8)	40,880,000
South-East				
	Abia	161,906 (5.0)	163,240 (5.0)	3,269,000
	Anambra	212,657 (4.4)	330,448 (6.9)	4,819,000
	Ebonyi	138,351 (5.9)	206,495 (8.8)	2,345,000
	Enugu	97,200 (2.6)	261,480 (7.0)	3,717,000
	Imo	186,002 (4.2)	349,292 (7.9)	4,402,000
Subtotal		667,299 (3.6)	1,310,955 (7.1)	18,552,000
South-South				
	Akwa Ibom	238,460 (5.3)	163,240 (3.7)	4,461,000
	Cross River	199,383 (5.7)	129,921 (3.7)	3,472,000
	Bayelsa	101,747 (4.9)	63,079 (3.0)	2,087,000
	Rivers	157,512 (2.7)	22,927 (0.4)	5,759,000
	Delta	128,923 (2.7)	136,775 (2.9)	4,747,000
	Edo	121,167 (3.2)	142,754 (3.8)	3,804,000
Subtotal		947,192 (3.9)	607,609 (2.5)	24,330,000
South-West				
	Ekiti	455,419 (1.8)	195,280 (7.8)	2,516,000
	Lagos	128,945 (0.9)	25,696 (0.2)	14,480,000
	Ogun	121,225 (3.1)	109880 (2.8)	3,953,000
	Ondo	117,752 (3.2)	176578 (4.8)	3,679,000
	Osun	80,635 (2.0)	181,129 (4.4)	4,105,000
	Oyo	98,555 (1.5)	24,512 (0.4)	6,532,000
Subtotal		598,937 (1.7)	713,075 (2.0)	35,265,000
Sum Total		3,276,360 (2.0)	8,682,068 (5.3)	163,818,000

*Abbreviations*: ICT, immunochromatographic test, LF, lymphatic filariasis, MDA, mass drug administration, Mf, microfilaria

## Discussion

Maps produced in this analysis are intended to estimate the prevalence of LF in unsampled locations, highlight intra-district heterogeneity of infection, and estimate the population infected with LF. These would help guide programme activities for a more focussed intervention. Here, our results are particularly helpful in classifying LGAs in Borno State which are yet to be mapped under the national mapping survey.

The predicted prevalence levels of LF ICT and Mf presented here demonstrate that LF distribution in Nigeria is largely ubiquitous. For ICT, prevalence estimates were highest in the North-Central (8.2%), North-West (7.8%), and South-East (7.1%) regions, and lowest in the South-South (2.5%) region. In contrast, prevalence estimates for Mf were generally higher in the southern than in the northern regions. Overall, predicted mean national prevalence was 5.3% and 2.0% for ICT and Mf, respectively. As MDA for LF is ongoing in Nigeria, robust estimates of baseline prevalence are important for evaluating the efficiency of control efforts.

Models provided state-level LF prevalence predictions for Nigeria, delineating within-region heterogeneities in infection prevalence. The ICT prevalence estimates generated from this analysis are in keeping with earlier work describing the environmental suitability of LF in Nigeria (Additional file 2: Figure S2) [28]. Both maps also correspond well with the distribution of *Anopheles* spp., which is the chief vector for LF transmission in Nigeria [29] and endemicity maps of the national LF control programme [2]. The climate and environmental variables that contributed the most to predicting the prevalence of LF in unsampled locations were precipitation, land surface temperature, and distance to permanent water bodies. The influence of these variables on the mosquito vectors and their effect in driving the transmission of LF has been discussed in our previously published work [28]. Machine learning methods have been widely used to model distribution of various parasitic diseases, both for species classification [45] or for regression analysis [34]. Their ability to handle non-linear associations between response and predictor variables, control for interactions among predictor variables and handle large complex datasets is a major advantage of these methods [34].

In this work, the prevalence of LF in Nigeria was modelled using the QRF algorithm which is an extension of the RF. The models were constructed by combining infection prevalence data from both ICT and Mf diagnostic types; however, final maps were projected according to diagnostic type. The main reason for the uneven availability of ICT and Mf data is that the ICT-based survey is considered the method of choice for the mapping of LF prior to intervention, and has been used for this purpose since 2000. In contrast, Mf surveys were conducted only in areas suspected to be highly endemic for LF, using lymphedema and hydrocele cases as an indicator for high endemicity. This left large portions of the country without Mf survey points, with some states with no survey points at all, while others were very sparsely surveyed. A visual observation of the Mf survey plot shows more dense clustering of the surveys in the southern parts of the country. This selective sampling of sites may have biased the Mf survey locations as hard-to-reach and more rural areas are more likely to be ignored. Also, as blood testing is performed at night (between 10 pm and 2 am) to coincide with the nocturnal periodicity of the parasite in blood, this cumbersome approach may be a contributing factor to a biased and selective survey.

The ICT and Mf prevalence predictions presented in this work are distinctly different. It is well known, however, that estimates for ICT are generally higher than Mf estimates even in surveys conducted in similar locations [5]. Understanding this contrasting prevalence values is an ongoing challenge in LF research and previous works have attempted to model the relationship between ICT and Mf prevalence [46, 47]. Irvine et al. [46] demonstrated that ICT and Mf prevalence is a consequence of the distribution of adult worms and the subsequent microfilariae production, although it is suggested that ICT prevalence is relatively uninformative in providing estimates of the infective pool [46]. This is mainly due to the therapeutic action of MDA and the nonlinear relationship between adult worm burden and Mf output [46]. Mf prevalence provides a more accurate estimate of microfilariae worm load and thus, a good tool for measuring infectious pool within LF endemic communities.

Furthermore, the treatment regimen used for MDA is known to be more effective against the microfilariae and less so for the adult worms [48]. Therefore, following treatment, ICT prevalence tend to decline more slowly due to the continued presence of the adult worms and their production of the filarial antigen which is still detected in blood using ICT cards [48]. Mf prevalence declines much more quickly due to the stronger microfilaricidal effects of treatment. In addition, in Nigeria, where there are vast areas co-endemic for both LF and onchocerciasis [49], and of which MDA for onchcocerciasis, using ivermectin, pre-dates the survey data used in this study. Although this study models the baseline prevalence of LF, it does not account for the therapeutic effects of onchocerciasis treatment in LF co-endemic areas [50].

Although ICT and Mf prevalence maps are contrasting, this is not to say that one is better or that predictions from the other should be disregarded. Overall, the main determinant factor in the QRF model for the LF prevalence maps is the diagnostic type (Fig. 3), indicating that this accounts for most of the variability in distribution. Understandably, ICT and Mf prevalence maps appear quite different from each other. However, as mentioned earlier, the main goal of the ICT surveys is to better understand the general geographical extent of infection transmission [7], while the Mf surveys, due to the rapid microfilaricidal effects of treatment, are useful for providing a more precise numerical measure with which to evaluate control interventions and track control progress.

The predictive accuracy of models is usually tested by exploring the ability of the model to correctly predict on an independent dataset [51]. As there was no independent dataset to test the model on, predictive accuracy was evaluated by calibrating the QRF model on a random sample of 75%, and then predicting on the held-out 25%. Predictive accuracy was measured by the value of the *R*^2^, which is the percentage of variation explained by the climate and environmental covariates included in the model. With an *R*^2^ value of 40%, more than half of the variation in the model predictions is not explained by factors included in our analysis. In this work, effects of the malaria control programmes (particularly bednet usage) and its influence on LF prevalence in areas of co-endemicity were not accounted for. This is perhaps a factor to consider going forward, as malaria and LF endemic areas largely overlap [52] and bednets used for malaria prevention have been widespread in Nigeria [53] and have been demonstrated to be protective against LF [54]. Further, with the increase in gross domestic product of Nigeria by more that 10-fold (from $46.4 billion in 2000 to $514.96 billion in 2013) [55], it is believed that general living conditions are improved, providing better protection against mosquito vectors [56]. A larger proportion of the population living above the poverty line and better awareness of the aetiology of LF and malaria may also have led to personal protection measures from the mosquito vectors.

Although the random forest algorithm is growing in popularity for use for spatial predictions, it fails to account for residual spatial correlations in observations [57], however, the inclusion of corresponding geographical coordinates of the survey dataset as a predictor could address this issue. The existence of spatial autocorrelation on the cross-validation residuals is an indication of suboptimal model predictions [57]. Our results (Fig. 6) suggest no evidence of spatial autocorrelation in the cross-validation residuals.

After more than five rounds of MDA, the North-Central states of Plateau and Nasarawa have demonstrated evidence of interruption of transmission and in 2017 transmission assessment survey commenced [58]. For large portions of the country that MDA is currently ongoing, findings from this work will aid re-assessment of programme activities. For instance, in the allocation of preventive chemotherapy and making sure that number of treatments offered are enough to achieve the stipulated population and programme coverage. Furthermore, there is a risk of resurgence in areas where transmission has been interrupted mainly due to the continued presence of mosquito vectors and within-country human migration. Areas previously identified as highly endemic for LF will be key in monitoring prevalence levels going forward. Additionally, periodic entomological examination of mosquito vectors for the presence of the filarial antigen (xenomonitoring) is an effective tool to determine whether the parasite is still present in populations where transmission had been interrupted [59]. This should be used in combination with Mf and ICT surveys, though caution must be exercised when evaluating control measures by testing with ICT because filarial antigenemia is still detected in blood samples long after MDA has been completed [6].

Maps are presented in this work have relatively wide prediction intervals. Given these wide intervals, predictions are in keeping with previous knowledge of LF endemicity in Nigeria [60]. However, there are several ways to narrow intervals in QRF models. It has been suggested that decreasing the spatial resolution reduces uncertainty [38]. Lower resolution maps may be more useful for predicting infections at a larger geographical scale (for instance, continent-wide or on a global scale) as is intended to give estimates for regions rather than smaller areas within a country. Here maps have been projected at a 5 × 5 km spatial resolution in order to better delineate intra-state prevalence levels. Maps produced at lower resolution will be a lot coarser and risk losing their intended purpose of estimating prevalence levels at the lowest administrative level. However, mapped estimates of the mean prevalence estimates for ICT closely mirror maps describing the ecological niche of LF in Nigeria (Additional file 2: Figure S2) [28] and it is believed these prevalence estimates are a fair picture of the LF distribution for Nigeria.

The human population infected with LF in Nigeria was previously estimated to be 13.53 million [10]. This estimate, however, was derived from modelling 27 infection data points of which the most recent survey was conducted in the year 1990 using population estimates of the year 2000. These surveys were mostly conducted by individual researchers in their region of interest with no inter-survey standardisation. The geographical extent and standardised protocol of the survey dataset used for the present analysis coupled with a robust modelling method provides the most comprehensive picture of LF endemicity in Nigeria to date. We however did not account for age structure in our population estimates; therefore, figures presented in this work are likely to be higher than the actual infected population.

Mathematical models have been used to assess the impact of intervention (mainly MDA and vector control) on the LF transmission [61]. The three most commonly used models for evaluating LF interventions are the population-based model, EPIFIL [62], and individual-based models TRANSFIL and LYMFASIM [63, 64]. These models have been trialled on data from LF endemic communities in India, Papua New Guinea and Kenya [61]. One key parameter in these models is baseline LF prevalence levels. These prevalence levels, however, were obtained from surveys conducted by in-country programmes which were sometimes patchy and did not have complete geographical coverage of the area of interest. Further, these data follow the structure of RAGFIL mapping, where entire districts are classed as either endemic or non-endemic according to results from a single survey point within the district. Estimates from our work, however, provide a comprehensive picture of the baseline LF burden for Nigeria. This will be invaluable data to parameterise these models in different settings and to assess the spatial heterogeneity of control efforts.

## Conclusions

Modelling the baseline endemicity of infection should ideally be performed prior to scaling up control programmes. Good knowledge of the extent of disease burden is also useful for raising awareness and serves as framework for advocacy for community/institutional engagement. Since the LF control programme is already ongoing in Nigeria, these model estimates provide a basis with which to evaluate control efforts and encourage more coordination towards reaching the elimination targets. Prevalence estimates provided may also serve as a proxy for estimating the burden LF morbidity (lymphedema and hydrocele) [65] in line with planning morbidity management and disability prevention programmes.

## Supplementary information

**Additional file 1: Figure S1.** Cross-validation of the predicted lymphatic filariasis prevalence using a subsample of 25% of the observed data. Predicted prevalence values are plotted against observed prevalence. Those plotted in red had observations outside of the 95% prediction intervals while those plotted in blue had observations inside the 95% prediction intervals.

**Additional file 2: Figure S2. a** Mean predicted prevalence of lymphatic filariasis in Nigeria. **b** Predicted environmental suitability of lymphatic filariasis in Nigeria [28].

## Data Availability

The datasets used for this work are available in the Expanded Special Project for Elimination of Neglected Tropical Diseases (ESPEN) repository http://espen.afro.who.int/countries/nigeria. predictor datasets and R code are available upon reasonable request to the corresponding author.

## Abbreviations

GLSM: generalised linear spatial model
ICT: immunochromatographic test
IU: implementation unit
LF: Lymphatic filariasis
LGA: local government area
MDA: mass drug administration
Mf: microfilaria
QRF: quantile regression forest
RAGFIL: Rapid Assessment of the Geographical Distribution of Bancroftian Filariasis
RF: random forest
RMSE: root mean square error
