# Supplementary figures and images for: Mapping the baseline prevalence of lymphatic filariasis across Nigeria
Source: Parasit Vectors. 2019 Sep 16;12:440. doi: 10.1186/s13071-019-3682-6 (PMC6745770; doi:10.1186/s13071-019-3682-6)

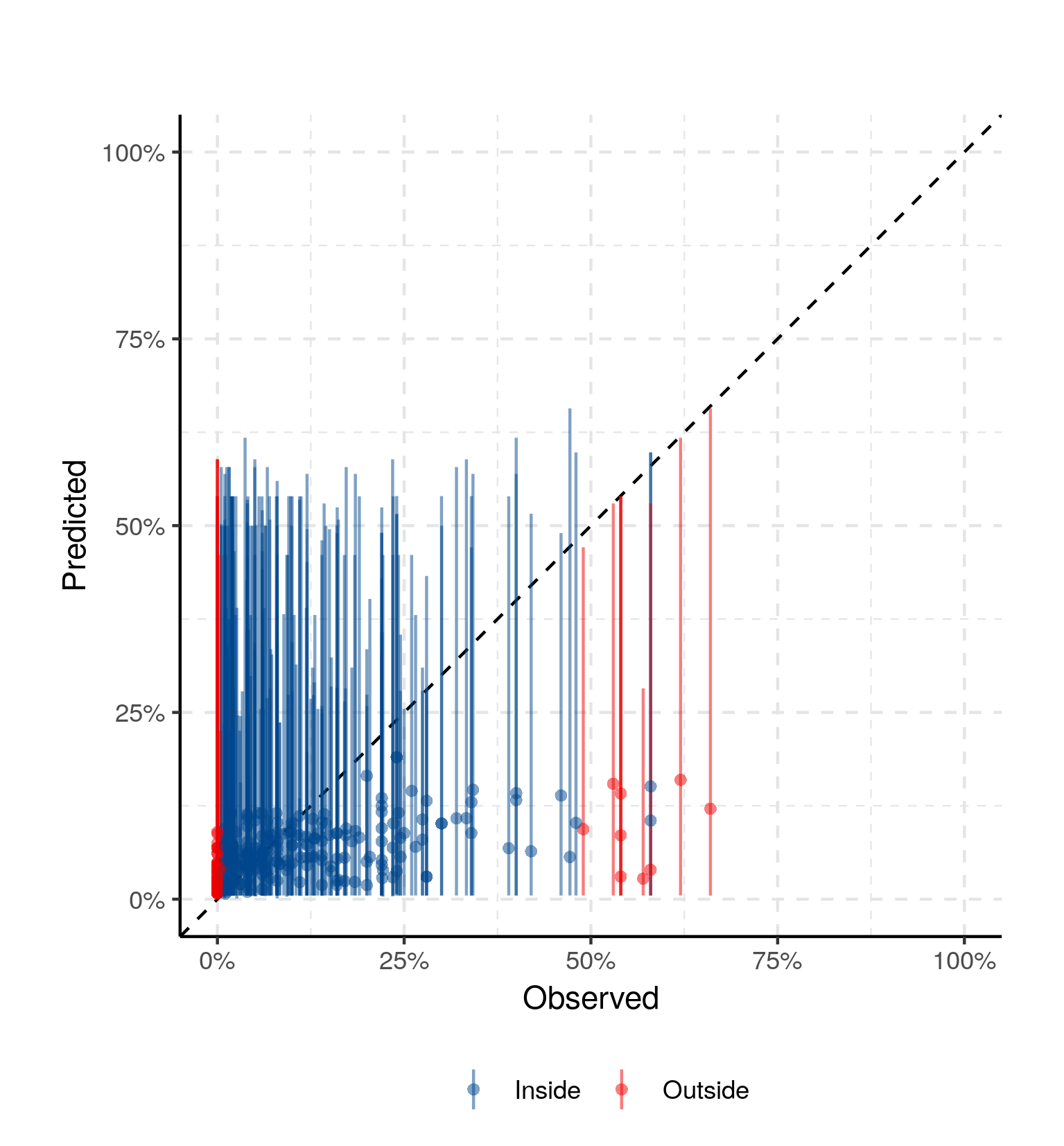

Supplement: Supplementary file 1 — Additional file 1: Figure S1. Cross-validation of the predicted lymphatic filariasis prevalence using a subsample of 25% of the observed data. Predicted prevalence values are plotted against observed prevalence. Those plotted in red had observations outside of the 95% prediction intervals while those plotted in blue had observations inside the 95% prediction intervals. [file 13071_2019_3682_MOESM1_ESM.tiff]

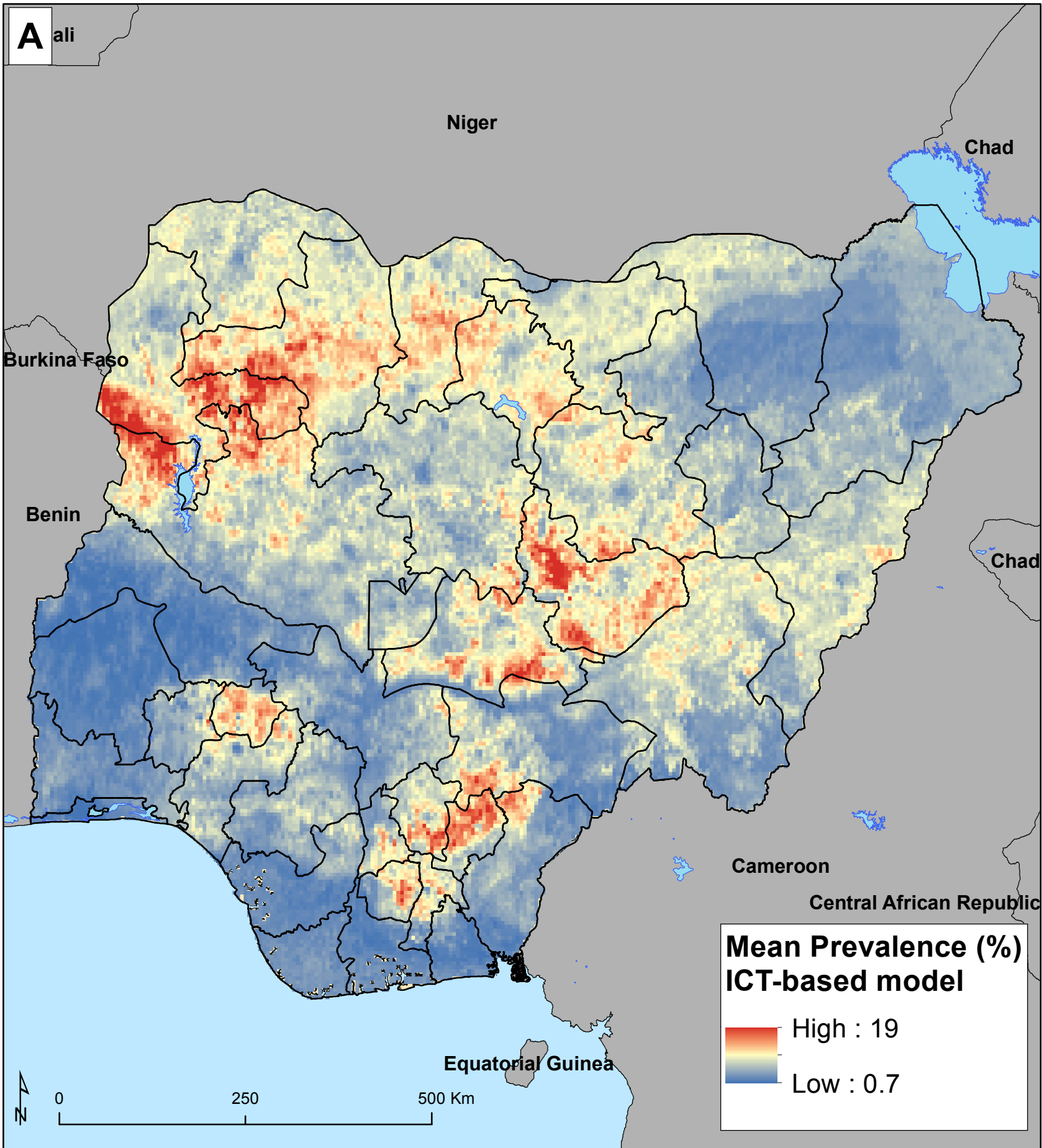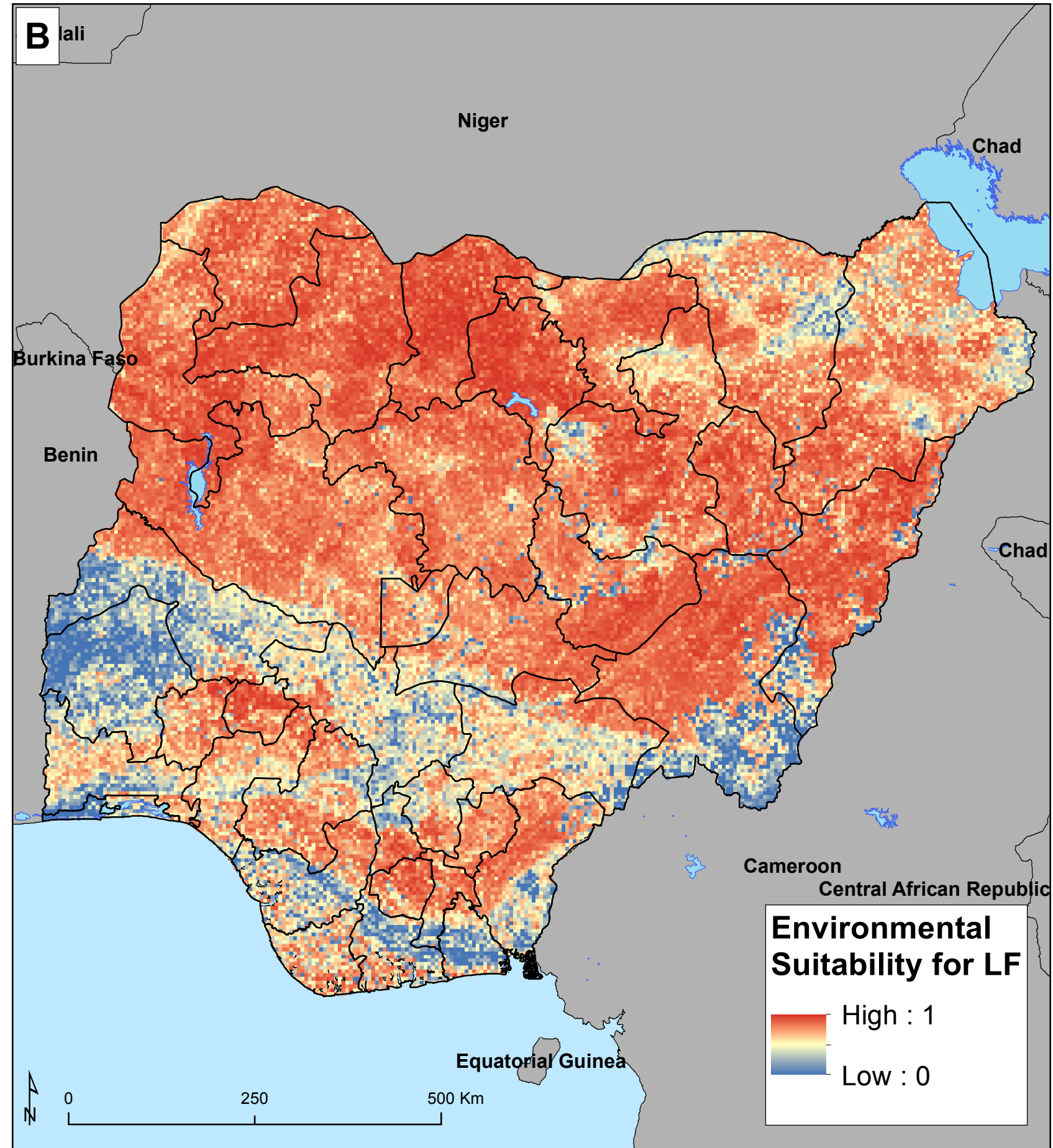

Supplement: Supplementary file 2 — Additional file 2: Figure S2. a Mean predicted prevalence of lymphatic filariasis in Nigeria. b Predicted environmental suitability of lymphatic filariasis in Nigeria [28]. [file 13071_2019_3682_MOESM2_ESM.pdf]
